# Bilateral Exertional Compartment Syndrome With Endoscopic Fasciotomy Surgical Intervention in a High School Athlete

**DOI:** 10.7759/cureus.13327

**Published:** 2021-02-13

**Authors:** Madeleine C Gwinn, Aaron McGuffin

**Affiliations:** 1 Pediatrics, West Virginia School of Osteopathic Medicine, Lewisburg, USA

**Keywords:** bilateral compartment syndrome, endoscopic fasciotomy, exertional compartment syndrome

## Abstract

A 17-year-old female presented to the physical therapy clinic with bilateral lower leg pain that worsened with activity. The patient experienced numbness, tingling, and cramping along the lateral and posterior portions of her legs during basketball practice, and her symptoms had gradually worsened over the past eight months. She obtained minimal relief with conservative treatments such as stretching and rest. X-rays and MRI of the lower limbs were obtained six months after symptoms began and were unremarkable. Further evaluation included compartment pressure testing taken before and after exercise. The patient demonstrated diagnostic pressures indicative of compartment syndrome in two compartments bilaterally. The patient was subsequently diagnosed with exertional compartment syndrome.

Exertional compartment syndrome is a cause of muscle pain that occurs due to increased tissue pressure within the confinement of the closed fascial space during exercise. Patients are often misdiagnosed or there is a significant delay in the correct diagnosis. The gold standard for diagnosis is measurement of intracompartmental pressures with the Stryker catheter.

Clinicians should consider exertional compartment syndrome in active patients who present with generalized muscle pain or sensation deficits that worsen with activity and are relieved with rest. Surgical intervention is a reasonable intervention and the only definitive option for an athlete with chronic exertional compartment syndrome who wants to continue high-level competition. Endoscopic fasciotomies are the new preferred techniques compared to more invasive open surgeries, which require a full incision. Endoscopic fasciotomy has a quicker healing time and has been shown to be as effective at relieving compartment syndrome symptoms as invasive open techniques.

After surgical intervention, the patient reported a 90% reduction in symptoms and had returned to full sport participation within two months.

## Introduction

Compartment syndrome is a well-known condition requiring immediate attention due to increased intracompartmental pressures, which can lead to vascular compromise and limb ischemia. However, exertional compartment syndrome is less widely known and often an overlooked diagnosis. Consequently, this patient case serves as an important learning tool to improve recognition of the symptoms associated with chronic exertional compartment syndrome and the surgical interventions available for resolution. We will discuss the case of a 17-year-old female athlete whose symptoms were significantly limiting her athletic performance. We will also discuss the steps that were taken to diagnosis and treat her condition.

This study was previously presented as a poster at the WVSOM (West Virginia School of Osteopathic Medicine) Alumni Association Mid-Winter Conference, January 25, 2020, Charleston, West Virginia, USA.

## Case presentation

A healthy 17-year-old female presented to the clinic with bilateral lower leg pain that worsened with activity. Symptoms began as a gradual aching and cramping along the lateral and posterior portions of her legs. Increased weight-bearing exercises, specifically running, caused increased symptoms including sensation deficits in her lower legs. Symptoms progressed to the point where the patient was unable to play continuously. She required several non-weight-bearing breaks and lower extremity stretching to continue with activity. At the height of her symptoms, post-exercise, her physical examination revealed decreased dorsiflexion, generalized weakness, and decreased sensation in the lateral and posterior lower leg. Gait analysis showed a “tip toe gait” with decreased stride length and absent heel strike during deceleration. Posterior tibial and dorsalis pedis pulses were normal. Sensation returned to normal as soon as the patient became non-weight-bearing. Early intervention included stretching, phonophoresis ultrasound with 1% dexamethasone, bilateral orthotic shoe placement, NSAIDs, and heat/ice therapy. However, all conservative interventions failed to relieve symptoms during exercise.

Diagnostic methods

X-rays of her tibia, fibula, and ankle mortise along with an MRI of her bilateral lower limbs were unremarkable. Further diagnostic studies included compartment pressure testing. Pressures were taken 10 minutes prior to running on a treadmill and immediately afterwards.

Table 1 summarizes the compartment pressure testing pre- and post-exercise. The patient met the diagnostic criteria in two compartments pre-exercise and in four compartments post-exercise.

**Table 1:** Compartment testing results pre- and post-exercise

Compartment	Pressure Readings (mmHg) Pre-Exercise	Pressure Readings (mmHg) Post-Exercise
Right anterior	9	25
Right lateral	20	27
Right posterior superficial	2	9
Right posterior deep	7	8
Left anterior	11	28
Left lateral	18	25
Left posterior Superficial	0	4
Left posterior deep	1	7

A resting pressure of 15 mmHg and post exercise pressure 20 mmHg are diagnostic of chronic compartment syndrome [1].

## Discussion

The waxing and waning presentation with activity makes the diagnosis of chronic compartment syndrome difficult. The diagnosis relies heavily on the patient history and timing of symptoms, which adds to the complexity of the diagnosis. Classical symptoms are pain in the affected compartment at the same time, distance, or intensity of exercise [2]. Typical diagnosis of chronic compartment syndrome has a 22-month delay [3]. Other differentials are more common and require less invasive diagnostics. These include medial tibial stress syndrome, stress fractures, other nerve entrapment syndromes, and deep vein thrombosis. However, if there is strong suspicion for chronic exertional compartment syndrome, the diagnosis should be pursued.

The lower leg is divided into four compartments: anterior, lateral, superficial posterior, and deep posterior. The tibialis posterior muscle is contained within its own fascia and is considered the fifth compartment of the leg [3].

**Figure 1:**
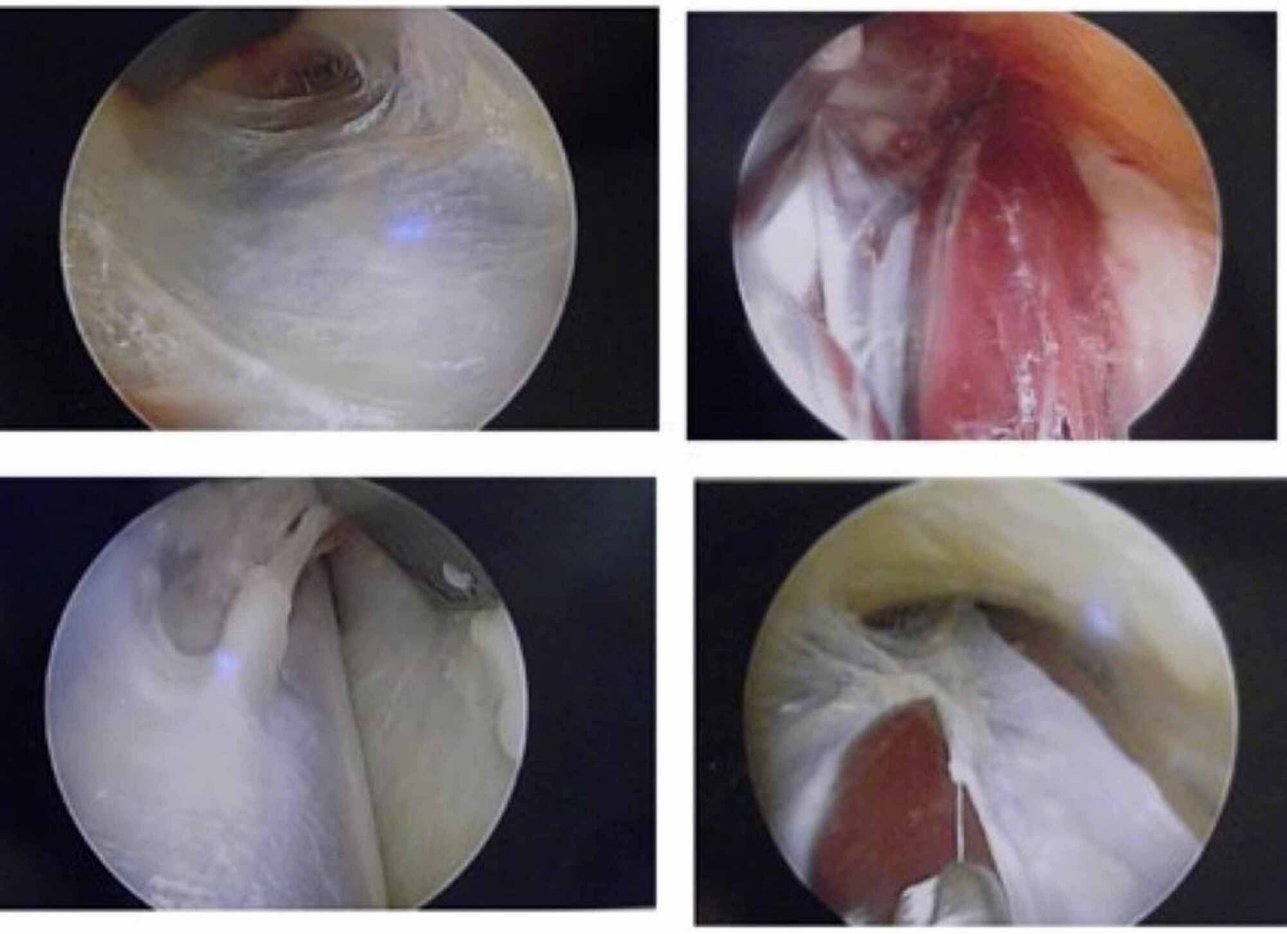
Top left: anterior compartment before fasciotomy. Top right: anterior compartment after fasciotomy. Bottom left: lateral compartment before fasciotomy. Bottom right: lateral compartment during fascia release.

Figure 1 operation imaging demonstrates the anterior compartment and the lateral compartment before and after the surgical fasciotomy.

Exertional compartment syndrome is a cause of muscle pain that occurs due to increased tissue pressure within the confinement of a closed fascial space during exercise [2]. The muscle volume can increase up to 20% of its resting size during exercise. This increase in muscle volume increases the internal pressure within the fascial compartment [1]. The pain caused by this increase in internal pressure is thought to be related to impaired tissue perfusion [4]. The gold standard of diagnosing chronic exertional compartment syndrome is the measurement of intracompartmental pressures. The Stryker catheter is the most widely used instrument for pressure testing. The procedure includes placing a needle inside one of the four compartments and injecting normal saline into that compartment. The pressure of the compartment is then taken and measured in mmHg [2].

The patient elected to undergo bilateral endoscopic fasciotomy to the anterior, lateral, and posterior compartments. Endoscopic fasciotomy is a subcutaneous technique that is less invasive than open fasciotomy. This technique is favored due to the smaller incision and quicker healing time [5]. Anterior and lateral fasciotomies have the best outcome with a greater than 80% success rate [6]. Overall complication of this intervention is 11-13% and includes hemorrhage, infection, nerve damage, deep vein thrombosis, vascular injury, skin breakdown, lymphocele, altered sensation over the fasciotomy site, and nerve entrapment [7].

**Figure 2:**
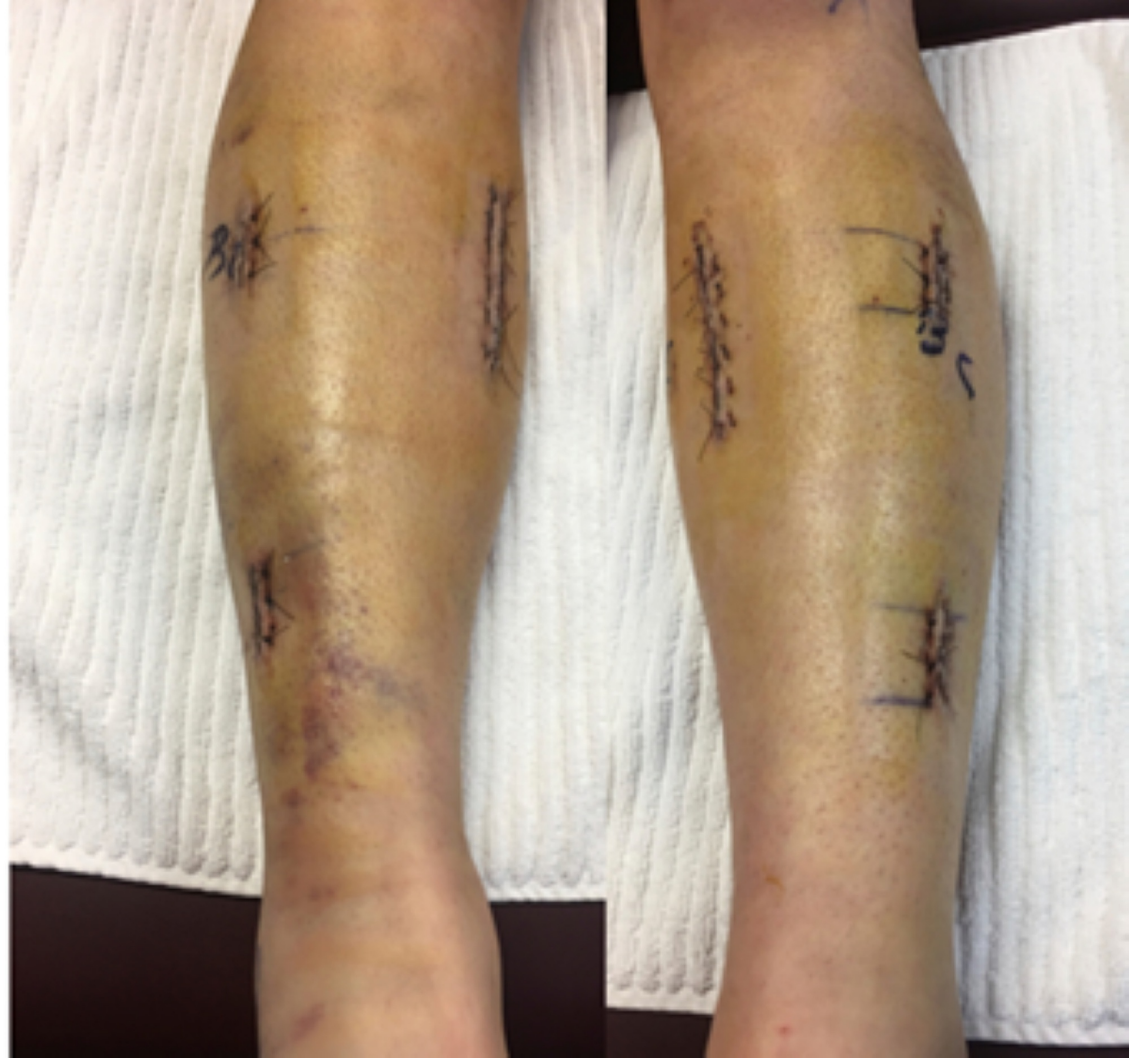
Patient incisions 24 hours after surgery.

Figure 2 illustrates the small incisions that were used for the endoscopic procedure.

## Conclusions

Once the incisions were healed, the patient began a return to play protocol, which included a mobility and strengthening plan. Within two months of surgery, she returned to full participation in basketball, no longer exhibited gait abnormalities, and reported 90% resolution of symptoms.

Exertional compartment syndrome should be considered in the differential when an active patient presents with generalized muscle pain or sensation deficits that are relieved by rest. MRI and X-rays should be completed to rule out other etiologies. Compartment pressure testing is the gold standard for the diagnosis of chronic exertional compartment syndrome. A surgical fasciotomy is commonly the treatment of choice because conservative treatments often do not fully resolve symptoms. An athlete can return to full activity in six to eight weeks if symptom-free and have recovered full strength and flexibility, making this an ideal treatment option.
